# Perceived organizational support and psychological capital in the relationship between job crafting and occupational wellbeing in CSSD nurses: a cross-sectional study

**DOI:** 10.3389/fpubh.2026.1799155

**Published:** 2026-09-04

**Authors:** Yanhong Wang, Liping He, Qing Xia

**Affiliations:** 1 Department of CSSD, Children's Hospital of Chongqing Medical University, National Clinical Research Center for Children and Adolescents' Health and Diseases, Ministry of Education Key Laboratory of Child Development and Disorders, Chongqing, China; 2 Chongqing Key Laboratory of Child Neurodevelopment and Cognitive Disorders, Chongqing, China; 3 Department of Burn and Plastic Surgery, Children's Hospital of Chongqing Medical University, National Clinical Research Center for Children and Adolescents' Health and Diseases, Ministry of Education Key Laboratory of Child Development and Disorders, Chongqing, China; 4 Department of Neurology, Children's Hospital of Chongqing Medical University, National Clinical Research Center for Children and Adolescents' Health and Diseases, Ministry of Education Key Laboratory of Child Development and Disorders, Chongqing, China

**Keywords:** central sterile supply department, job crafting, nurse, occupational wellbeing, perceived organizational support, psychological capital

## Abstract

**Objective:**

To examine the indirect associations of perceived organizational support and psychological capital in the relationship between job crafting and occupational wellbeing among nurses working in central sterile supply departments (CSSDs).

**Methods:**

Between April and May 2025, a total of 401 CSSD nurses were recruited using convenience sampling from seven provinces in China. Data were collected using a general information questionnaire, the Perceived Organizational Support Scale, the Psychological Capital Questionnaire, the Job Crafting Scale, and the Occupational Wellbeing Scale.

**Results:**

The mean scores were as follows: perceived organizational support (61.53 ± 10.07), psychological capital (82.43 ± 10.46), job crafting (47.17 ± 7.44), and occupational wellbeing (86.70 ± 9.05). Female CSSD nurses reported lower occupational wellbeing scores compared with their male counterparts. Nurses with more than 20 years of total work experience in CSSDs exhibited lower occupational wellbeing scores than those with 10 years of experience. Path analysis revealed that job crafting had a weak direct association on occupational wellbeing (β = 0.185, 95% CI = 0.026–0.308, *P* < 0.001), whereas the total effect was substantial (0.838). Indirect associations were observed through perceived organizational support (β = 0.511, 95% CI = 0.417–0.644, *P* < 0.001) and psychological capital (β = 0.142, 95% CI = 0.060–0.226, *P* = 0.001).

**Conclusion:**

Perceived organizational support and psychological capital showed significant indirect associations in the relationship between job crafting and occupational wellbeing among CSSD nurses. The indirect associations are hypothesis-generating and require confirmation in longitudinal or experimental studies before intervention recommendations can be made.

## Introduction

The Central Sterile Supply Department (CSSD) is a critical unit in hospital infection control, responsible for cleaning, disinfecting, sterilizing, and supplying sterile medical instruments (1). With increasing precision in surgical equipment and ongoing advances in medical technology, CSSD nurses encounter heavier workloads, significant physical demands, and limited opportunities for career advancement (2). As a subjective experience shaped by individuals' cognitive evaluations and emotional responses to work-related factors, occupational wellbeing directly influences work engagement, quality of care, and workforce retention (3). Therefore, enhancing occupational wellbeing among CSSD nurses has emerged as a critical concern for healthcare management.

Multiple stressors contribute to diminished wellbeing in this population. CSSD work is frequently undervalued, and many nurses perceive a disconnect between their efforts and the recognition or rewards they receive, resulting in a weakened sense of professional value (4). Unlike many bedside nursing roles, CSSD work involves repetitive high-precision tasks, substantial infection-control responsibility, limited direct patient feedback, and relatively low professional visibility, making organizational and personal resources particularly relevant to occupational wellbeing. These occupational characteristics highlight the urgent need to identify effective strategies to enhance occupational wellbeing in this essential workforce.

Job crafting, defined as employees' proactive modifications of tasks, relationships, and perceptions of work, is a key behavioral factor that may enhance wellbeing (5). Previous studies have demonstrated that job crafting can increase job satisfaction and foster positive work outcomes among nurses (6). However, the potential indirect associations linking job crafting to occupational wellbeing remain insufficiently explored. Grounded in the Job Demands-Resources (JD–R) theory, which posits that job resources (e.g., organizational support) and personal resources (e.g., psychological capital) play critical roles in buffering job demands and promoting wellbeing (7), this study further examines potential indirect pathways. According to the JD–R theory, proactive work behaviors such as job crafting enable employees to optimize job resources and better cope with work demands, thereby promoting occupational wellbeing. This theoretical framework supports the expected positive association between job crafting and occupational wellbeing.

Perceived organizational support and psychological capital may also serve as important behavioral factors influencing wellbeing. A study involving 1,831 Chinese nurses found a strong positive correlation between perceived organizational support and occupational wellbeing (*r* = 0.685). Higher levels of perceived organizational support were associated with greater occupational wellbeing (8). Sheng et al. reported that perceived organizational support significantly moderated the relationship between the practice environment and wellbeing; under conditions of high organizational support, the practice environment had a significant positive effect on wellbeing (β = 0.38) (9). This relationship can also be explained by Social Exchange Theory, which suggests that employees who perceive stronger organizational support are more likely to reciprocate through positive work attitudes and enhanced wellbeing. Therefore, perceived organizational support may represent a potential indirect pathway in the association between job crafting and occupational wellbeing (10). In addition, a meta-analysis revealed an inverse correlation between psychological capital and both burnout and emotional exhaustion (11). Similarly, the Conservation of Resources theory, psychological capital represents an important personal resource that enables individuals to better cope with workplace stressors and maintain positive psychological functioning (12). This suggests that psychological capital may represent another potential indirect pathway in the association between job crafting and occupational wellbeing. Accordingly, the JD–R theory provides the overarching framework for the hypothesized parallel path model, whereas Social Exchange Theory and Conservation of Resources theory explain the organizational-support and psychological-capital pathways, respectively.

Taken together, these findings suggest that job crafting, perceived organizational support, and psychological capital are all closely associated with occupational wellbeing. Building on these empirical findings and theoretical perspectives, the present study proposes the following hypothesized parallel path model. Nonetheless, how perceived organizational support and psychological capital function in the relationship between job crafting and occupational wellbeing remains insufficiently understood, especially among CSSD nurses. Therefore, the present study aims to investigate whether job crafting is indirectly associated with occupational wellbeing through perceived organizational support and psychological capital among CSSD nurses. Within the JD–R framework, job crafting may be positively associated with occupational wellbeing by helping nurses adjust their work and access relevant resources. Social Exchange Theory supports an indirect association through perceived organizational support, whereas Conservation of Resources theory supports an indirect association through psychological capital. Accordingly, one direct and two parallel indirect associations were hypothesized.

H1: Job crafting is positively associated with occupational wellbeing among CSSD nurses.

H2: Job crafting is indirectly associated with occupational wellbeing through perceived organizational support.

H3: Job crafting is indirectly associated with occupational wellbeing through psychological capital.

## Methods

### Participants

Participants were recruited from 35 hospitals across seven provincial-level regions in southwestern China (Sichuan, Chongqing, Guizhou, and Yunnan), central China (Hubei and Henan), and eastern China (Shandong). Nurses in CSSDs were recruited from these hospitals between April and May 2025. To protect participant anonymity, hospital names and other institution-level identifiers were not recorded in the questionnaire.

Inclusion criteria were as follows: (1) registered nurses with at least 1 year of work experience in a CSSD; and (2) voluntary participation with informed consent. Exclusion criteria included: (1) nurses on leave (vacation, personal leave, or maternity leave) during the survey period; and (2) nurses temporarily assigned to the CSSD for advanced training, rotation, or standardized residency training who were not regular CSSD staff.

This study was approved by the Ethics Committee of Children's Hospital of Chongqing Medical University [Approval No. 118, (2025)].

### Study design

A cross-sectional survey design was employed. Initially, the head nurses of CSSDs in participating hospitals were contacted and provided with detailed information about the background, objectives, and significance of the study. Upon obtaining their approval, the head nurses introduced the study to eligible nurses using standardized instructions, emphasizing the voluntary and anonymous nature of participation.

Data were collected anonymously through the Wenjuanxing online survey platform. All questionnaire items were mandatory and presented as single-choice questions. Based on pilot testing and the estimated time required to complete all questionnaire items, responses completed in less than 180 s were considered potentially invalid due to insufficient time for careful reading and answering, whereas responses exceeding 3,000 s were considered likely to reflect interruptions or inattentive completion.

## Measures

### General information questionnaire

The research team developed a self-designed questionnaire to collect participants' demographic and occupational characteristics. These included gender, age, professional title, years of service, job position, night-shift rotation, marital status, childbearing status, hospital level, income, and related variables.

### Perceived organizational support scale

The Perceived Organizational Support Scale, developed by Wenquan et al. (13), consists of 16 items across three dimensions: work support, value recognition, and concern for wellbeing. Responses are rated on a 5-point Likert scale ranging from 1 (“strongly disagree”) to 5 (“strongly agree”), with total scores ranging from 16 to 80. Higher scores indicate greater perceived organizational support. In the present study, the Cronbach's α coefficient was 0.944, indicating excellent internal consistency.

### Psychological capital questionnaire

The Psychological Capital Questionnaire, adapted into Chinese by Luo et al. in 2010 (14), assesses four dimensions: self-efficacy, optimism, resilience, and hope. It employs a 6-point Likert scale ranging from 1 (“strongly disagree”) to 6 (“strongly agree”). Items 13 and 20 are reverse-scored. Higher total scores reflect higher levels of psychological capital. In the present study, Cronbach's α coefficient was 0.939, indicating high internal consistency.

### Job crafting questionnaire

The Job Crafting Questionnaire, developed by Slemp et al. (15) in 2013, is a three-dimensional scale that includes task crafting, relational crafting, and cognitive crafting. The reliability coefficients of the subscales range from 0.83 to 0.87. The Chinese version was validated by Kui et al. (16). In this study, the Cronbach's α coefficient was 0.943.

### Occupational wellbeing scale

Occupational wellbeing was assessed using the scale developed by Hu et al. (17), which comprises (17) five dimensions: value realization, physical and mental health, social support, work environment, and economic income. Each item is rated on a 5-point Likert scale, with total scores ranging from 24 to 120. Higher scores indicate greater occupational wellbeing. Cronbach's α coefficients of the subscales ranged from 0.815 to 0.895, demonstrating good internal consistency.

### Sample size

The required sample size was estimated based on the commonly recommended guideline for structural equation modeling (SEM), which suggests a minimum sample size of 10 times the number of parameters to be estimated. In this study, the primary focus was on the relationships among four scales. Considering the number of scale dimensions, the estimated number of parameters was approximately 20–30. Accounting for a 10%−20% rate of invalid responses, the minimum required sample size was calculated to be 360 CSSD nurses.

This study further employed Mplus (Muthén & Muthén, Los Angeles, CA, USA) 8.3 software to conduct Monte Carlo simulations to evaluate the adequacy and robustness of direct effects, indirect effects, total indirect effects, differences in indirect effects, and total effects under the current sample size. Results were considered satisfactory when the absolute value of relative bias in parameter estimation was less than 10%, the 95% confidence interval coverage ranged between 0.91 and 0.98, and statistical power exceeded 0.8.

### Statistical analysis

Normally distributed continuous variables were expressed as mean ± standard deviation (SD). Between-group comparisons were conducted using independent-samples *t*-tests and analysis of variance (ANOVA), with pairwise comparisons performed using the Student–Newman–Keuls (*q*) test. Pearson correlation coefficients were calculated to assess the strength of associations between variables. Common method bias was assessed by comparing a single-factor model, the hypothesized multifactor measurement model, and an unmeasured latent method construct (ULMC) model in which all items additionally loaded onto a common method factor. Measurement validity was evaluated using confirmatory factor analysis (CFA). Given that all study variables were measured using five-point Likert scales, first-order and second-order CFA models were estimated in Mplus using the Weighted Least Squares Mean and Variance Adjusted (WLSMV) estimator. Reliability and convergent validity were assessed separately for both first-order and second-order constructs using standardized factor loadings, composite reliability (CR), and average variance extracted (AVE), while measurement reliability was assessed using Cronbach's alpha. Discriminant validity between perceived organizational support and occupational wellbeing was further assessed using the Fornell–Larcker criterion and constrained CFA.

Modifications to the hypothesized cross-sectional path model were guided by modification indices (MI). Model fit was considered acceptable when χ^2^/df < 5.0, RMSEA < 0.08, TLI ≥ 0.90, CFI ≥ 0.90, and SRMR < 0.05. Modification indices were examined post hoc. Residual covariances were added only when MI >10 and when theoretically justifiable. Bias-corrected bootstrap testing with 5,000 resamples was employed to calculate 95% confidence intervals (CIs) for the direct and indirect associations. Structural equation modeling was conducted using Mplus 8.3, and all other analyses were performed in SAS 9.4 (SAS Institute Inc., USA). A two-tailed *P* value < 0.05 was considered statistically significant.

## Results

### Scores of job crafting, psychological capital, perceived organizational support, and occupational wellbeing among CSSD nurses and measurement model evaluation

A total of 401 CSSD nurses who met the inclusion criteria were included in the final analysis. Of the 427 questionnaires collected, 401 were deemed valid after data cleaning, resulting in an effective response rate of 93.9%.

The mean scores of the participating CSSD nurses were as follows: job crafting (total: 47.17 ± 7.44), psychological capital (total: 82.43 ± 10.46), perceived organizational support (total: 61.53 ± 10.07), and occupational wellbeing (total: 86.70 ± 9.05) (Table 1).

**Table 1:** Descriptive statistics of job crafting, psychological capital, perceived organizational support, and occupational wellbeing among nurses in the central sterile supply department.

Scale/Dimension	Items	Mean ±SD	Average score ±SD
**Job crafting**	12	47.17 ± 7.44	3.93 ± 0.62
Task crafting	4	15.00 ± 2.90	3.75 ± 0.73
Relational crafting	4	15.86 ± 2.73	3.97 ± 0.68
Cognitive crafting	4	16.31 ± 2.54	4.08 ± 0.64
**Psychological capital**	20	82.43 ± 10.46	4.12 ± 0.52
Optimism	3	12.75 ± 1.97	4.25 ± 0.66
Resilience	5	20.15 ± 2.98	4.03 ± 0.60
Hope	6	24.57 ± 3.64	4.09 ± 0.61
Self-efficacy	6	24.97 ± 3.39	4.16 ± 0.56
**Perceived organizational support**	16	61.53 ± 10.07	3.85 ± 0.63
Work support	7	26.69 ± 4.40	3.81 ± 0.63
Value recognition	5	19.45 ± 3.30	3.89 ± 0.66
Concern for wellbeing	4	15.39 ± 3.24	3.85 ± 0.81
**Occupational wellbeing**	24	86.70 ± 9.05	3.61 ± 0.38
Physical & mental health	6	17.00 ± 5.52	2.83 ± 0.92
Value/ability realization	6	23.29 ± 4.08	3.88 ± 0.68
Social support	5	20.47 ± 2.87	4.09 ± 0.57
Economic income	3	10.33 ± 2.97	3.44 ± 0.99
Work environment	4	15.59 ± 2.94	3.90 ± 0.73

SD, standard deviation.

As shown in Table 2, all constructs demonstrated good reliability and validity. Measurement properties were evaluated separately for first-order and second-order constructs. Cronbach's α values ranged from 0.816 to 0.944, indicating satisfactory internal consistency. Standardized factor loadings for all first-order dimensions ranged from 0.664 to 0.981. In addition, CR values ranged from 0.832 to 0.968 for first-order constructs and ranged from 0.919 to 0.973 for second-order constructs, while AVE values ranged from 0.510 to 0.884 and from 0.696 to 0.923, respectively, all exceeding the recommended thresholds of 0.70 and 0.50.

**Table 2:** Reliability and convergent validity of first- and second-order constructs.

Construct/dimension	Items	Cronbach's α	Standardized factor loading of second-order CFA	CR	AVE	Square root of AVE
Job crafting	12	0.943	–	0.957	0.883	0.940
Task crafting	4	0.827	0.890	0.878	0.645	0.803
Relational crafting	4	0.871	0.981	0.918	0.738	0.859
Cognitive crafting	4	0.933	0.945	0.967	0.880	0.938
**Psychological capital**	20	0.939	–	0.965	0.874	0.935
Optimism	3	0.759	0.900	0.841	0.642	0.801
Resilience	5	0.687	0.929	0.832	0.510	0.714
Hope	6	0.902	0.959	0.940	0.724	0.851
Self-efficacy	6	0.884	0.950	0.928	0.683	0.826
**Perceived organizational support**	16	0.944	–	0.973	0.923	0.961
Work support	7	0.836	0.924	0.921	0.639	0.799
Value recognition	5	0.861	0.992	0.930	0.729	0.854
Concern for wellbeing	4	0.949	0.965	0.968	0.884	0.940
**Occupational wellbeing**	24	0.816	–	0.919	0.696	0.834
Physical & mental health	6	0.884	0.664	0.910	0.638	0.799
Value/ability realization	6	0.864	0.915	0.917	0.657	0.811
Social support	5	0.815	0.886	0.899	0.648	0.805
Economic income	3	0.895	0.774	0.944	0.849	0.921
Work environment	4	0.866	0.903	0.917	0.734	0.857

CR, composite reliability; AVE, average variance extracted.

### Occupational well-being among CSSD nurses

Female CSSD nurses reported significantly lower occupational wellbeing scores compared with their male counterparts (88.27 ± 14.49 vs. 95.29 ± 15.88, *P* = 0.023). In addition, nurses with more than 20 years of total work experience had significantly lower occupational wellbeing scores than those with 10 years or less (86.92 ± 12.89 vs. 91.59 ± 16.77, *P* < 0.05) (Table 3).

**Table 3:** Occupational wellbeing among nurses in the central sterile supply department.

Variable	*n* (%)	Occupational wellbeing score (Mean ±SD)	*t*/*F*	*P*
**Age (years)**			0.477	0.621
≤ 30	75 (18.70)	89.37 ± 16.30		
31–45	232 (57.86)	88.98 ± 14.64		
>45	94 (23.44)	87.43 ± 13.30		
**Gender**			2.289	0.023
Male	24 (5.99)	95.29 ± 15.88		
Female	377 (94.01)	88.27 ± 14.49		
**Years of service (Total)**			3.191	0.042
≤ 10	108 (26.93)	91.59 ± 16.77		
11–20	166 (41.40)	88.16 ± 14.24		
>20	127 (31.67)	86.92 ± 12.89		
**Years in CSSD**			0.057	0.944
≤ 3	102 (25.44)	89.09 ± 15.34		
4–10	192 (47.88)	88.63 ± 14.87		
>10	107 (26.68)	88.42 ± 13.66		
**Current job position**			1.556	0.200
Staff nurse	224 (55.86)	89.16 ± 15.34		
Quality control leader	57 (14.21)	84.98 ± 16.11		
Head nurse	78 (19.45)	90.14 ± 11.42		
Others	42 (10.47)	88.55 ± 13.77		
**Night-shift rotation**			−1.484	0.139
Yes	145 (36.16)	87.17 ± 16.40		
No	256 (63.84)	89.55 ± 13.52		
**Professional title**			1.937	0.123
Nurse	60 (14.96)	92.18 ± 16.63		
Nurse practitioner	110 (27.43)	88.73 ± 15.12		
Senior nurse	171 (42.64)	87.08 ± 14.29		
Chief nurse	60 (14.96)	89.72 ± 12.09		
**Type of employing institution**			2.151	0.118
Tertiary	272 (67.83)	89.54 ± 14.82		
Secondary	103 (25.69)	87.69 ± 14.37		
Other	26 (6.48)	83.81 ± 13.13		
**Marital status**			1.035	0.356
Married	333 (83.04)	89.10 ± 14.56		
Single	45 (11.22)	85.76 ± 16.40		
Divorced/Widowed	23 (5.74)	88.52 ± 11.99		
**Childbearing status**			1.684	0.187
No children	69 (17.21)	86.48 ± 15.53		
One child	228 (56.86)	88.50 ± 14.55		
Two or more children	104 (25.94)	90.59 ± 14.17		
**Monthly income (CNY)**			2.203	0.112
< 5,000	116 (28.93)	89.60 ± 15.50		
5,000–10,000	245 (61.10)	87.64 ± 14.63		
>10000	40 (9.98)	92.48 ± 11.36		
**Only-child status**			−1.705	0.089
Yes	124 (30.92)	86.83 ± 14.95		
No	277 (69.08)	89.52 ± 14.46		

No significant differences in occupational wellbeing scores were found across other demographic or occupational variables, including age, years of experience in the CSSD, current job position, night-shift rotation, professional title, employing institution, marital status, childbearing status, monthly income, or only-child status (*P* > 0.05) (Table 3).

### Correlation analysis of job crafting, psychological capital, perceived organizational support, and occupational wellbeing

**Figure 1:**
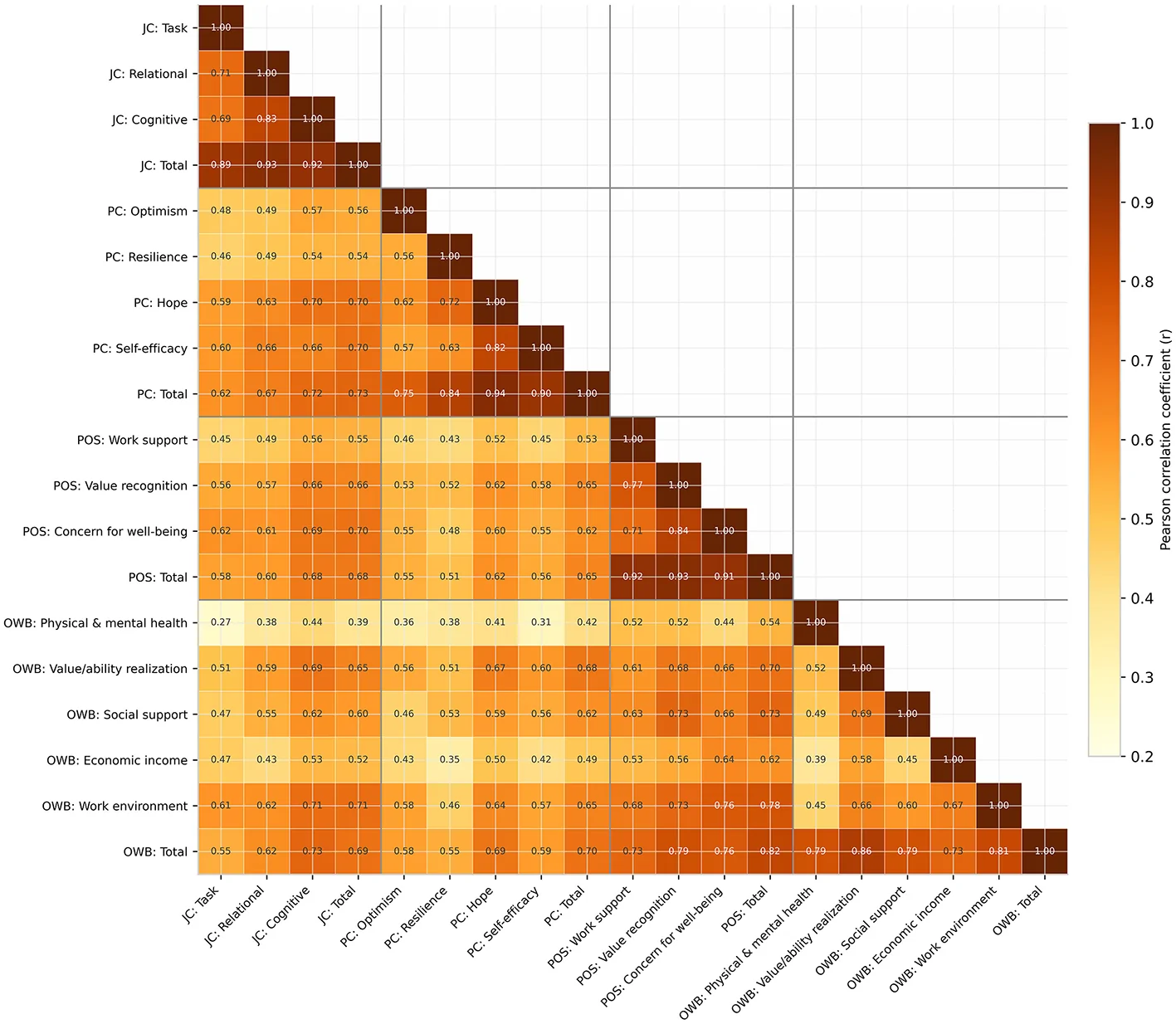
Heat map of Pearson correlation coefficients among job crafting, psychological capital, perceived organizational support, and occupational wellbeing among nurses in the central sterile supply department (*n* = 401); (all *P* < 0.05). JC, Job crafting; PC, Psychological capital; POS, Perceived organizational support; OWB, Occupational wellbeing).

Widespread positive associations were observed among job crafting, psychological capital, perceived organizational support, and occupational wellbeing. Specifically, all dimensions of job crafting—particularly cognitive crafting—were positively correlated with psychological capital, perceived organizational support, and occupational wellbeing. Among these dimensions, cognitive crafting showed the strongest correlations with the total scores of psychological capital (*r* = 0.72), perceived organizational support (*r* = 0.68), and occupational wellbeing (*r* = 0.73); (all *P* < 0.01) (Figure 1).

Within psychological capital, the hope dimension showed a strong correlation with occupational wellbeing (*r* = 0.69). Among the dimensions of perceived organizational support, value recognition showed the strongest correlation with occupational wellbeing (*r* = 0.79) (Figure 1). Collectively, the results provide a theoretical basis for designing interventions that target psychological capital and foster supportive organizational practices. Correlations between demographic variables and the main study variables were weak (|*r*| < 0.20; see Supplementary Table 1).

Given the strong correlation between perceived organizational support and occupational wellbeing (*r* = 0.82), their discriminant validity was further examined. The square roots of the AVE were 0.961 for perceived organizational support and 0.834 for occupational wellbeing, both exceeding the correlation between the two constructs (Supplementary Table 2). In the constrained CFA, fixing the latent correlation between the two constructs at 1.00 substantially worsened model fit compared with the freely estimated two-factor model (CFI: 0.795 vs. 0.899; RMSEA: 0.190 vs. 0.133). These findings suggest that perceived organizational support and occupational wellbeing were empirically distinguishable.

In addition, the single-factor model fitted the data poorly (χ^2^/df = 6.020, CFI = 0.833, TLI = 0.829, SRMR = 0.083, RMSEA = 0.122) compared with the hypothesized multifactor model (χ^2^/df = 3.292, CFI = 0.927, TLI = 0.922, SRMR = 0.052, RMSEA = 0.076). Adding a common method factor improved model fit (χ^2^/df = 1.862, CFI = 0.973, TLI = 0.971, SRMR = 0.034, RMSEA = 0.046). The results showed that the improvement in model fit was limited (ΔCFI = 0.046, ΔRMSEA = 0.030, both less than 0.05). Moreover, 56 of the 72 method-factor loadings (77.8%) were non-significant, and the mean absolute loading was 0.13, suggesting that method effects were not pervasive across items.

### Cross-sectional path analysis of occupational wellbeing, job crafting, psychological capital, and perceived organizational support among CSSD nurses

**Figure 2:**
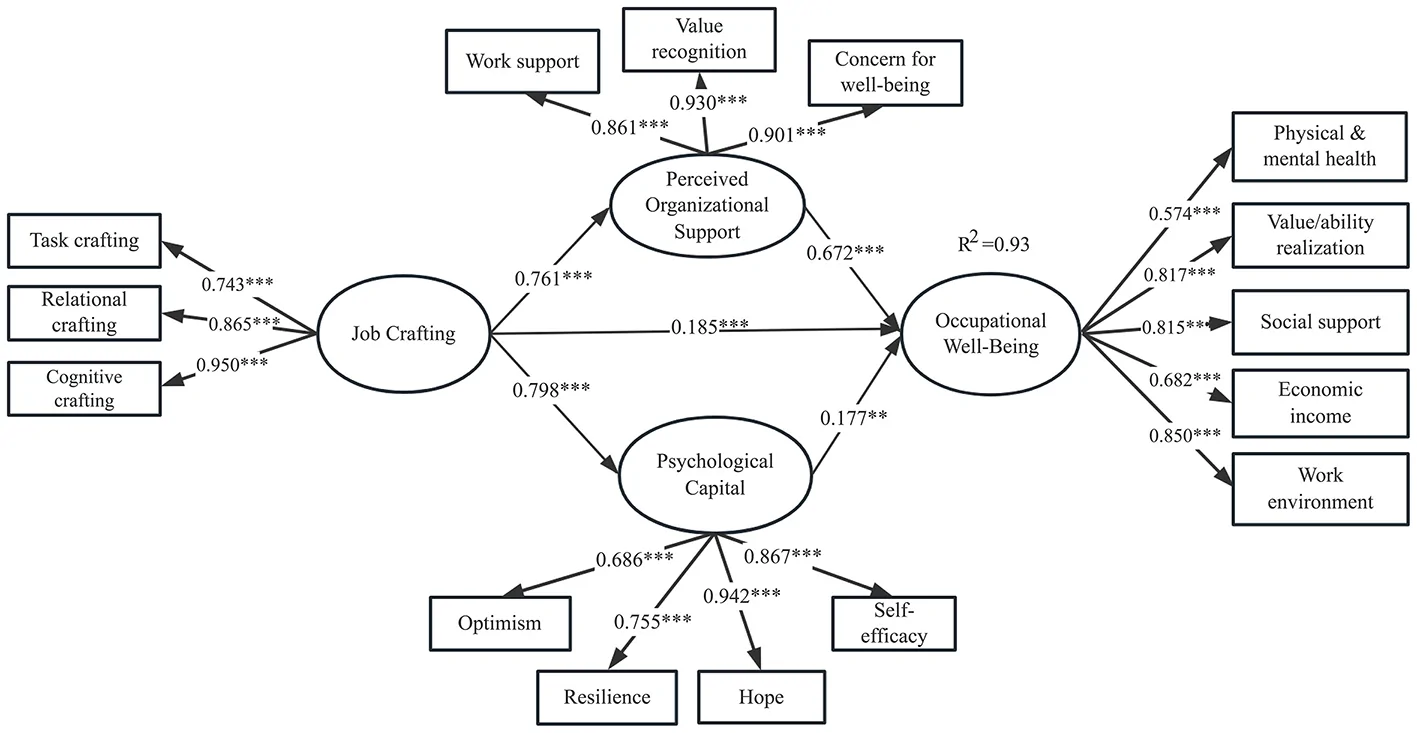
Cross-sectional path model of occupational wellbeing among nurses in the central sterile supply department (**P < 0.01; ***P < 0.001).

**Figure 3:**
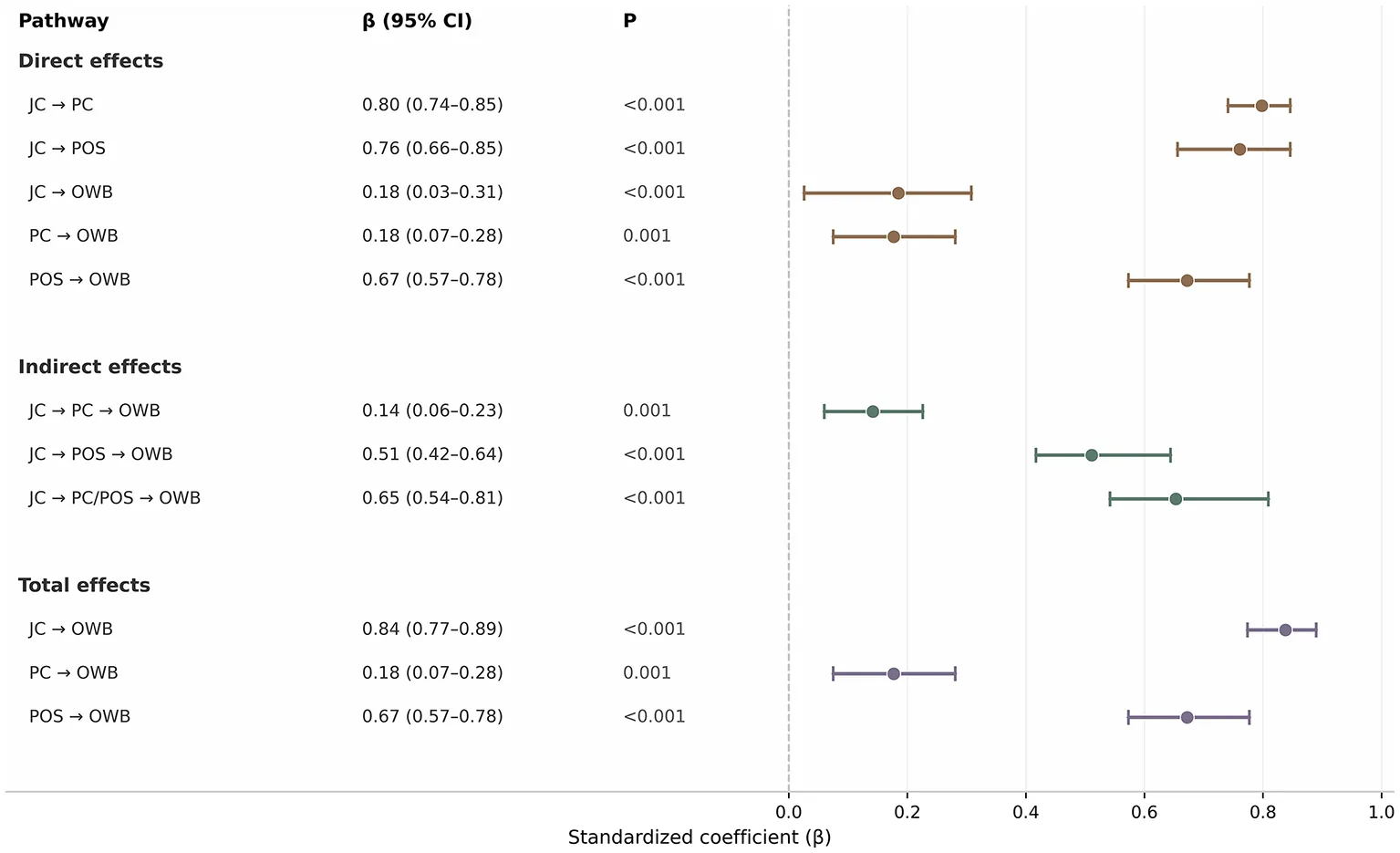
Forest plot of the standardized estimates for direct, indirect, and total associations of job crafting, psychological capital, and perceived organizational support on occupational wellbeing among nurses in the central sterile supply department. The 95% CI were calculated using 5,000 Bootstrap resamples. JC, Job crafting; PC, Psychological capital; POS, Perceived organizational support; OWB, Occupational wellbeing.

The initial model yielded χ^2^/df = 4.092, CFI = 0.948, TLI = 0.935, SRMR = 0.039, and RMSEA = 0.088. Four residual covariances were added: task crafting with relational crafting (MI = 10.41), social support with economic income (MI = 16.14), social support with work environment (MI = 18.59), and economic income with work environment (MI = 29.35). The modified cross-sectional path model (Figure 2) showed improved fit (χ^2^/df = 3.502, CFI = 0.960, TLI = 0.941, SRMR = 0.037, RMSEA = 0.079), without material changes in the structural-path estimates. The direct, indirect, and total effects on occupational wellbeing are summarized in Figure 3.

Job crafting, psychological capital, and perceived organizational support were all directly associated with occupational wellbeing, with direct effect estimates of 0.185 (95% CI = 0.026–0.308, *P* < 0.001), 0.177 (95% CI = 0.075–0.281, *P* = 0.001), and 0.672 (95% CI = 0.573–0.777, *P* < 0.001), respectively. Among the direct pathways, perceived organizational support showed the strongest effect. Although the direct association between job crafting and occupational wellbeing was modest (β = 0.185), the total association was larger (β = 0.838), with indirect associations observed through perceived organizational support and psychological capital. Although statistically significant, the relatively small coefficient suggests limited practical impact when considered in isolation. Among the two indirect associations examined, the association through perceived organizational support [0.511 (95% CI = 0.417–0.644), *P* < 0.001] was larger than that through psychological capital [0.142 (95% CI = 0.060–0.226), *P* = 0.001]. A formal contrast confirmed this difference (unstandardized difference = 0.543, 95% CI: 0.327–0.809).

### Monte carlo simulation

At *N* = 401 and based on 2,000 replications, the Monte Carlo simulation showed that the simulated mean estimates of the path coefficients and effect parameters were broadly consistent with their corresponding population values. The absolute relative parameter biases ranged from 0.042% - 0.119%, remaining below the prespecified threshold of 10%, and the mean squared errors ranged from 0.002-0.014. Empirical 95% CI coverage ranged from 0.943 - 0.955, within the prespecified acceptable range of 0.91–0.98. Power ranged from 0.942 - 1.000 for all tested direct, specific indirect, total indirect, indirect-effect contrast, and total effects, with all power estimates exceeding 0.80. This included the pathway corresponding to the standardized specific indirect effect of 0.142. Thus, all examined effects met the prespecified Monte Carlo criteria under the fitted model (Table 4).

**Table 4:** Monte Carlo simulation results of direct, indirect, and total effects of job crafting, psychological capital, and perceived organizational support on occupational wellbeing among nurses in the central sterile supply department.

Path	Truth-value in SEM	Monte carlo simulation				
	Unstandardized coefficient	Estimated value	Relative deviation (%)	Mean square error	95% cover	Power
Direct effects						
JC → PC	0.500	0.501	0.042	0.002	0.951	1.000
JC → POS	1.269	1.275	0.098	0.010	0.951	1.000
JC → OWB	0.272	0.272	0.079	0.006	0.955	0.942
PC → OWB	0.416	0.418	0.116	0.013	0.947	0.963
POS → OWB	0.592	0.596	0.061	0.004	0.947	1.000
Indirect effects						
JC → POS → OWB	0.751	0.758	0.089	0.008	0.943	1.000
JC → PC → OWB	0.208	0.209	0.059	0.003	0.947	0.963
JC → PC/POS → OWB	0.959	0.967	0.111	0.013	0.948	1.000
JC → POS → OWB vs JC → PC → OWB	0.543	0.549	0.101	0.010	0.947	1.000
Total effects						
JC → OWB	1.231	1.239	0.119	0.014	0.950	1.000

JC, job crafting; PC, psychological capital; POS, perceived organizational support; OWB, occupational wellbeing.

## Discussion

This study identified differences in occupational wellbeing across gender and work experience among CSSD nurses, with female nurses and those with longer service duration reporting lower wellbeing. These patterns may reflect cumulative occupational stress and gender-related vulnerabilities in demanding healthcare environments. The relatively weak direct association suggests that job crafting alone was not strongly associated with occupational wellbeing when considered independently of perceived organizational support and psychological capital. These findings highlight the strong association between perceived organizational support and occupational wellbeing. To the best of our knowledge, few studies have simultaneously examined the associations, highlighting the incremental contribution of this study.

Existing studies consistently report that the burden of anxiety, depression, and sleep disorders is significantly higher among female healthcare workers than among male healthcare workers. Given that females constitute the majority of the nursing workforce, nurses are more likely to experience moderate to severe anxiety and depression compared with physicians (18). Moreover, a 24-year prospective cohort study of 46,318 US female nurses found that longer durations of rotating night shift work were associated with a lower probability of healthy aging (19). These findings indicate that prolonged occupational exposure exerts cumulative adverse effects on the physical and psychological health of female nurses, which may partly explain the lower occupational wellbeing scores observed among women in this study.

In addition, research conducted by Chen et al. in low-risk regions of China revealed that nurses with 11–15 years of work experience had significantly higher levels of anxiety and depression, indicating that the mid-career stage may represent a critical period for psychological vulnerability (20). Similarly, Serafin et al. reported that the beneficial effect of psychological resilience on job satisfaction is most pronounced during the early stage of a nursing career, whereas senior nurses tend to depend more on external resources such as professional competence and social support (21). Collectively, these findings suggest that the occupational wellbeing of nurses does not decline linearly with years of service but instead encounters greater challenges during the mid-career stage, with psychological protective mechanisms evolving dynamically across different career phases. Therefore, when designing mental health intervention strategies, it is crucial to consider stage-specific characteristics of work experience and to pay particular attention to the psychological needs of mid-career nurses.

Path analysis indicated that job crafting was associated with occupational wellbeing both directly and indirectly through perceived organizational support and psychological capital. An Australian survey found that psychological wellbeing is positively associated with workplace resilience and higher education levels, highlighting the importance of proactive adjustments at both individual and organizational levels (22). Similarly, a Finnish quasi-experimental study provided the first evidence that a 6-week online job crafting intervention significantly improved job crafting behaviors and work engagement among public sector employees, including nurses, while also exerting a protective effect on autonomic nervous function (23). Collectively, these findings suggest that education and institutional support warrant further evaluation as potential approaches to enhancing occupational wellbeing.

Of the two indirect associations examined, the association through perceived organizational support was larger than that through psychological capital, highlighting the potential relevance of organizational resources. O'Connor et al. reported that organizational strategies such as professional development opportunities, reflective practices, and leadership support can effectively strengthen professional competence and psychological resilience of nurses (24). Supporting this, Cooper et al. found that nurses who participated frequently (≥75%) in peer support groups exhibited significant reductions in depression, stress, and emotional exhaustion, indicating a positive correlation between intervention frequency and wellbeing (22). However, Cooper et al. also emphasized that if structural work pressures—such as staffing shortages or scheduling conflicts—remain unresolved, interventions may be perceived as an additional burden, thereby diminishing their effectiveness. Consequently, organizational support must be institutionalized, with managerial commitment to ensuring adequate time and resources, to achieve sustained improvements in the wellbeing of nurses.

In addition to the indirect association through perceived organizational support, the hypothesized model showed another indirect association through psychological capital (β = 0.142). During public health emergencies, nurses' general self-efficacy not only directly enhances their professional identity but also exerts a substantial indirect effect through psychological resilience, with the mediating pathway accounting for 75.16% of the total effect (21). This finding suggests that self-efficacy enhances resilience, thereby reinforcing professional identity. Likewise, the trait of hope has been shown to promote participation in professional training and innovative practices by enhancing professional commitment (β = 0.41) (21). Consistently, a qualitative study of nurses in COVID-19 intensive care units in Wuhan reported a 27% reduction in job burnout among those who set phased recovery goals and adjusted their implementation strategies accordingly (25). Collectively, these findings suggest that positive psychological resources—such as hope and self-efficacy—warrant further investigation in longitudinal and intervention studies of nurses' occupational wellbeing.

In the hypothesized model, perceived organizational support as a job resource and psychological capital as a personal resource both showed indirect associations between job crafting and occupational wellbeing. These findings represent an application, rather than an extension, of the JD–R framework. The observed associations suggest that job crafting, perceived organizational support, and psychological capital may warrant further investigation as potential intervention targets. However, the present cross-sectional findings do not provide sufficient evidence to recommend specific workplace strategies. Longitudinal and experimental studies are needed to determine whether modifying these factors can improve occupational wellbeing among CSSD nurses.

This study has several limitations. First, the cross-sectional design precluded establishing temporal ordering or causal mediation, and reverse or bidirectional associations remain possible. Second, convenience sampling across seven Chinese provinces and the absence of hospital identifiers limited assessment of sample representativeness and hospital-level clustering. Unaccounted within-hospital dependence may have underestimated the standard errors, and generalizability beyond similar healthcare settings should therefore be approached cautiously. Third, no a priori power analysis was conducted for the specific indirect effects, and the Monte Carlo simulation was retrospective. Fourth, although the instruments showed satisfactory internal consistency, they were not specifically validated among CSSD nurses. Concurrent self-report measurement may also have introduced common method variance, while the strong correlation between perceived organizational support and occupational wellbeing raises the possibility of conceptual overlap despite evidence of discriminant validity. Fifth, gender and total years of service were not included as covariates, leaving potential residual confounding. Finally, the post hoc, data-driven model modifications require validation in an independent sample.

## Conclusion

In this cross-sectional sample of CSSD nurses, job crafting, perceived organizational support, psychological capital, and occupational wellbeing were positively associated. In the hypothesized parallel path model, job crafting showed indirect associations with occupational wellbeing through both perceived organizational support as an organizational resource and psychological capital as a personal resource. However, the study cannot determine whether job crafting preceded changes in these resources or occupational wellbeing, and reverse or bidirectional associations remain possible. Therefore, the findings do not provide sufficient evidence to recommend specific workplace interventions. Longitudinal studies with repeated measurements are needed to establish temporal ordering, followed by intervention studies to determine whether modifying job crafting, organizational support, or psychological capital improves occupational wellbeing among CSSD nurses.

## Data Availability

The raw data supporting the conclusions of this article will be made available by the authors, without undue reservation.
