# Supplementary material for: Perceived organizational support and psychological capital in the relationship between job crafting and occupational wellbeing in CSSD nurses: a cross-sectional study
Source: Front Public Health. 2026 Sep 4;14:1799155. doi: 10.3389/fpubh.2026.1799155 (PMC13585916; doi:10.3389/fpubh.2026.1799155)
Supplement: Supplementary file 1 [file Table_1.docx]

Supplementary Table 1. Correlations between gender, years of service, and psychological capital, perceived organizational support, and occupational well-being

| **Variable** | Gender (Male vs Female) | |  | Years of Service | |
| --- | --- | --- | --- | --- | --- |
|  | **r** | ***P*** |  | **r** | ***P*** |
| Psychological Capital | -0.161 | 0.001 |  | -0.021 | 0.674 |
| Perceived Organizational Support | -0.113 | 0.023 |  | -0.099 | 0.048 |
| Occupational Well-Being | -0.114 | 0.023 |  | -0.112 | 0.025 |
