# Supplementary material for: Perceived organizational support and psychological capital in the relationship between job crafting and occupational wellbeing in CSSD nurses: a cross-sectional study
Source: Front Public Health. 2026 Sep 4;14:1799155. doi: 10.3389/fpubh.2026.1799155 (PMC13585916; doi:10.3389/fpubh.2026.1799155)
Supplement: Supplementary file 2 [file Table_2.docx]

Supplementary Table 2. Discriminant validity of the second-order constructs assessed using the Fornell–Larcker criterion

| Construct/Dimension | Job Crafting | Psychological Capital | Perceived Organizational Support | Occupational Well-Being |
| --- | --- | --- | --- | --- |
| Job Crafting | 0.940 |  |  |  |
| Psychological Capital | 0.732 | 0.935 |  |  |
| Perceived Organizational Support | 0.679 | 0.648 | 0.961 |  |
| Occupational Well-Being | 0.694 | 0.699 | 0.823 | 0.843 |
